# The Use of the International Academy of Cytology Yokohama System for Reporting Breast Fine-Needle Aspiration Biopsy: A Systematic Review and Meta-Analysis

**DOI:** 10.1093/ajcp/aqac132

**Published:** 2022-11-12

**Authors:** Ilias P Nikas, Johannes A Vey, Tanja Proctor, Mousa M AlRawashdeh, Angela Ishak, Hyang Mi Ko, Han Suk Ryu

**Affiliations:** School of Medicine, European University Cyprus, Nicosia, Cyprus; Institute of Medical Biometry, University of Heidelberg, Heidelberg, Germany; Institute of Medical Biometry, University of Heidelberg, Heidelberg, Germany; School of Medicine, European University Cyprus, Nicosia, Cyprus; School of Medicine, European University Cyprus, Nicosia, Cyprus; Department of Laboratory Medicine and Pathobiology, University of Toronto, Toronto, Canada; Department of Pathology, Seoul National University College of Medicine, Seoul, Korea; Department of Pathology, Seoul National University Hospital, Seoul, Korea

**Keywords:** Cytopathology, Fine-needle aspiration, Reporting system, Breast cancer, Evidence-based medicine, Risk of malignancy, Sensitivity and specificity

## Abstract

**Objectives:**

To perform the first meta-analysis regarding the pooled risk of malignancy (ROM) of each category of the Yokohama system for reporting breast fine-needle aspiration, as well as assess the latter’s diagnostic accuracy using this new system.

**Methods:**

Two databases were searched, followed by data extraction, study quality assessment, and statistical analysis.

**Results:**

The “Insufficient,” “Benign,” “Atypical,” “Suspicious,” and “Malignant” Yokohama system categories were associated with a pooled ROM of 17% (95% CI, 10%-28%), 1% (95% CI, 1%-3%), 20% (95% CI, 17%-23%), 86% (95% CI, 79%-92%), and 100% (95% CI, 99%-100%), respectively. When both “Suspicious” and “Malignant” interpretations were regarded as cytologically positive, sensitivity (SN) was 91% (95% CI, 87.6%-93.5%) and false-positive rate (FPR) was 2.33% (95% CI, 1.30-4.14%). A summary receiver operating characteristic curve was constructed and the pooled area under the curve was 97.3%, while the pooled diagnostic odds ratio was 564 (95% CI, 264-1,206), indicating a high level of diagnostic accuracy. When only “Malignant” interpretations were regarded as cytologically positive, the pooled FPR was lower (0.75%; 95% CI, .39%-1.42%) but at the expense of SN (76.61%; 95% CI, 70.05%-82.10%).

**Conclusions:**

Despite Yokohama’s system early success, more data would be needed to unravel the system’s value in clinical practice.

**KEY POINTS**

- This is the first meta-analysis regarding the Yokohama system for reporting breast fine-needle aspiration (FNA).
- The pooled risks of malignancy of the “Benign” and “Malignant” interpretations were 1% and 100%, respectively.
- Reporting breast FNA with the Yokohama system was associated with high diagnostic accuracy.

## INTRODUCTION

Breast cancer has become the most common cancer worldwide, while it ranks fifth in the cancer mortality list, according to the latest GLOBOCAN estimates.^1^ Breast fine-needle aspiration (FNA) is a safe, minimally invasive, accurate, and cost-effective modality to assess palpable and nonpalpable breast lesions, which is best interpreted in correlation with the other components of the “triple test” (clinical examination and radiology), in a multidisciplinary context.^2-5^ However, it has largely been replaced by core-needle biopsy (CNB), especially in the setting of breast cancer screening programs.^3,6,7^ As a result, many laboratories have reduced the use of FNA only for specific applications—for instance, while evaluating cystic lesions or when the clinical suspicion of cancer is low.^6,8^

The International Academy of Cytology (IAC) Yokohama system for reporting breast FNA biopsy has been recently developed, having the goal to standardize reporting and improve communication among physicians.^2,3^ The system contains five reporting categories—“Insufficient,” “Benign,” “Atypical,” “Suspicious,” and “Malignant”^2,3^—each linked with a specific risk of malignancy (ROM) and a selection of management recommendations.^2,3^ As no meta-analysis has been performed so far regarding this new system, our study aimed to

1. Calculate the pooled ROM of each reporting category of the Yokohama system
2. Assess the diagnostic accuracy of breast FNA to detect malignancy, using the Yokohama system

## MATERIALS AND METHODS

### Search Strategy

This study was performed following the PRISMA guidelines.^9^ The PubMed and Scopus databases were searched for relevant studies until February 27, 2022, using the following search algorithm: (“Yokohama system” OR “International system” OR “International Academy of Cytology”) AND breast. The duplicate records were removed with the Paperpile reference manager (https://paperpile.com/app), while the PubMed search was updated on April 9, 2022.

### Study Selection

Study selection was first performed in a title-abstract fashion, using the Rayyan web tool (https://rayyan.ai/),^10^ followed by a full-text evaluation of the studies selected during the first step. Original breast FNA studies reporting their results with the Yokohama system were included in our review, when reporting 50 cases or more with available histopathologic and/or clinical follow-up. In contrast, studies using other reporting systems, reviews, editorials, case reports and small case series, and also studies written in a language other than English were excluded.

### Data Extraction

Data extraction was performed independently by three authors (I.P.N., A.I., and M.M.A.) in an independent fashion, while any disagreements were resolved by a consensus. The following data were extracted from each study: first author, year, country, study period, study type (retrospective vs prospective), use of image-guiding or rapid on-site evaluation (ROSE) during the FNA procedure, cytopreparation type, assessment of palpable vs nonpalpable lesions, follow-up type, and number of cases with follow-up. Furthermore, the numbers of total cases and events (carcinoma in situ or invasive cancer in follow-up) regarding each of the five Yokohama system categories were extracted from each study to calculate their pooled ROM. Last, to assess the diagnostic accuracy of breast FNA to detect cancer using the Yokohama system, we extracted the true-negative (TN), false-negative (FN), true-positive (TP), and false-positive (FP) cases from each study. The presence of carcinoma in situ or invasive cancer in histology was considered a positive (malignant) outcome. In addition, the few reported borderline and malignant phyllodes tumors were considered benign and malignant histologic outcomes, respectively. Furthermore, the results from the “Inadequate” Yokohama category were excluded for the accuracy analyses, while two different scenarios were followed in our data extraction. In scenario 1, “Suspicious” and “Malignant” cases found to be malignant in histology were extracted as TP values, whereas “Benign” and “Atypical” cases found to be malignant were extracted as FN values (Supplemental Table 1; all supplemental materials can be found at *American Journal of Clinical Pathology* online). In scenario 2, only “Malignant” cases were regarded as TP, whereas “Benign,” “Atypical,” and “Suspicious” cytology cases followed by malignant histology were regarded as FN (Supplemental Table 1).

### Study Quality Assessment

Study quality assessment was performed according to the domains proposed by the Quality Assessment of Diagnostic Accuracy Studies 2 (QUADAS-2) tool^11,12^ (Supplemental Table 2).

### Statistical Analysis

The statistical analysis was performed by two of the authors (J.A.V. and T.P.) using R, version 4.1.3 (R Foundation for Statistical Computing). To estimate the pooled ROM of each category of the Yokohama system, a random intercept logistic regression model was applied, and the between-study heterogeneity (τ^2^) was estimated using the maximum likelihood method. To calculate the confidence intervals (CIs) for the proportions, the Clopper-Pearson method was used. A continuity correction of 0.5 was applied in studies with zero cell frequencies. To assess the diagnostic accuracy of breast FNA cytology using the Yokohama system, summary receiver operating characteristic (sROC) curves were constructed, and the diagnostic odds ratio (DOR) was pooled in both scenarios. The sROC curves, their summary points (FP rate on the horizontal axis and sensitivity on the vertical axis), and the pooled area under the curve (AUC) value were estimated using the Rutter and Gatsonis^13^ hierarchical summary ROC model and the bivariate model described by Reitsma et al.^14^ The DORs were calculated from TP, TN, FP, and FN per study and were meta-analyzed applying a random-effects model using the inverse variance method and the restricted maximum likelihood estimator for τ^2^. In addition, a continuity correction of 0.5 was again applied in studies with zero cell frequencies. To identify potential reasons causing heterogeneity, selected subgroup and sensitivity analyses were performed according to year of publication, study design, reference test, and risk of bias judgment. Last, to assess potential publication bias, we used the method by Deeks et al,^15^ designed for diagnostic test accuracy studies.

## RESULTS

### Literature Search

**FIGURE 1:**
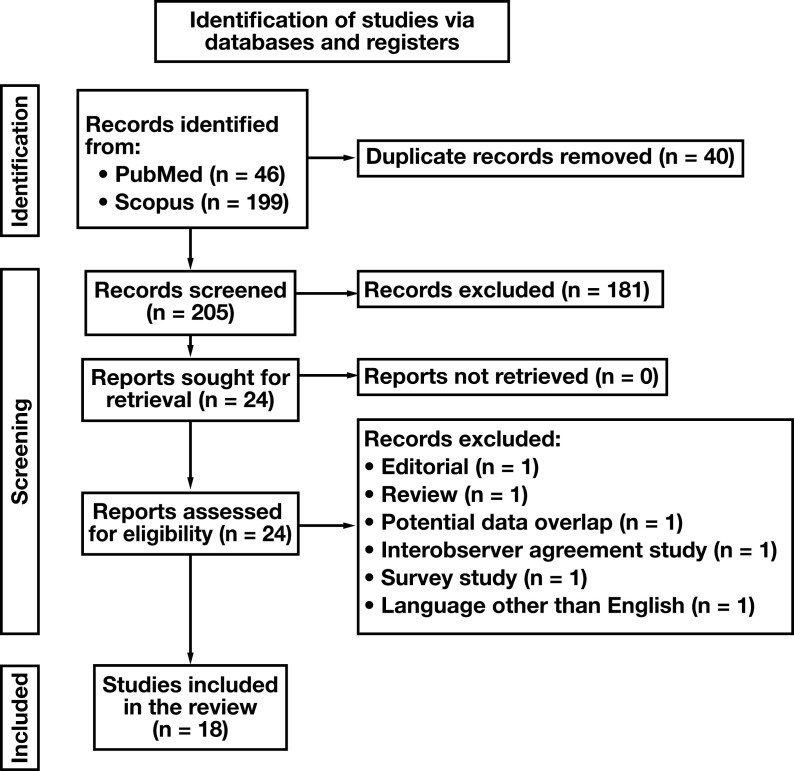
Flowchart of our study.

**FIGURE 1** shows the flowchart of our study. Initial search revealed 245 reports (46 in PubMed and 199 in Scopus). Duplicated records were removed, while title-abstract screening excluded 181 studies. Of the remaining 24 studies, 6 were further excluded while assessing their full text, resulting in 18 eligible studies for data extraction.^7,16-32^

### Study Characteristics

**TABLE 1** shows some characteristics of the studies included in our systematic review. Data from a total of 7,969 cases with follow-up were pooled. All 18 eligible studies were published between 2019 and 2021, and most of them (n = 11) were conducted in India. Most were retrospective (n = 16), whereas two^19,30^ followed a prospective design. None of the authors reported the sole use of liquid-based cytology as their cytopreparation method. Follow-up was based on histology in most studies (n = 16), whereas two^25,26^ used a combination of histology and clinical/radiologic follow-up. Three studies provided a separate data analysis using ROSE,^17,20,29^ and one study involved only male participants.^26^ Regarding the bias evaluation (Supplemental Table 2), two studies had a high bias risk under the “patient selection” domain—one of them selected patients with palpable masses and no radiologic guidance,^22^ whereas the other one included only males^26^—and two more under the domain “flow and timing,”^25,26^ as they did not use the same reference standard among the included patients.

**TABLE 1:** Main Characteristics of the Studies Included in This Meta-Analysis

First Author, Year	Country	Study Period	Study Type	Follow-up	No. of Cases With Follow-up
Nigam, 2021^16^	India	Jan 18–Dec 19	Retrospective	Histology	123
Agrawal, 2021^17^	India	Sep 16–Dec 18	Retrospective	Histology	624
Sundar, 2021^18^	India	Jan 16–Dec 20	Retrospective	Histology	287
Agrawal, 2021^19^	India	Sep 19–Nov 20	Prospective	Histology	487
Wong, 2021^20^	Malaysia	Jan 12–Dec 19	Retrospective	Histology	521
Tejeswini, 2021^21^	India	Jan 18–Jun 20	Retrospective	Histology	226
Sarangi, 2021^22^	India	Jan 16–Dec 19	Retrospective	Histology	400
Dixit, 2021^23^	India	Jul 16–Dec 18	Retrospective	Histology	285
Ahuja, 2021^24^	India	Jan 18–Dec 20	Retrospective	Histology	242
Marabi, 2021^25^	Hong Kong	Jan 01–Dec 16	Retrospective	Histology and clinical follow-up	1,012
Oosthuizen, 2020^26^	South Africa	Jan 15–Dec 19	Retrospective	Histology and clinical follow-up	82
Agarwal, 2020^27^	India	Sep 18–Aug 19	Retrospective	Histology	322
De Rosa, 2020^28^	Italy	2010-2017	Retrospective	Histology	1,745
Wong, 2019^29^	Australia	Jun 15–Feb 18	Retrospective	Histology	579
Panwar, 2019^30^	India	Jan 16–Jan 17	Prospective	Histology	108
Chauhan, 2019^31^	India	Apr 16–Mar 18	Retrospective	Histology	331
McHugh, 2019^32^	Kenya	Jan 99–Sep 17	Retrospective	Histology	219
Montezuma, 2019^7^	Portugal	Jan 07–Dec 17	Retrospective	Histology	776

### ROM of the Yokohama System Reporting Categories

**TABLE 2** shows the pooled ROM associated with each of the Yokohama system categories.

**TABLE 2:** Pooled Risk of Malignancy (ROM) Associated With Each International Academy of Cytology (Yokohama) System Category

Yokohama System Category	No. of Studies Pooled	ROM, %	95% CI, %	τsup>2/sup>	τ	*I*sup>2/sup>, %
Insufficient	18	17	10-28	1.2009	1.0959	81
Benign	18	1	1-3	1.2421	1.1145	73
Atypical	18	20	17-23	0.0370	0.1924	14
Suspicious	18	86	79-92	0.8739	0.9348	50
Malignant	18	100	99-100	2.6661	1.6328	17

The “Insufficient,” “Benign,” “Atypical,” “Suspicious,” and “Malignant” categories were associated with a pooled ROM of 17% (95% CI, 10%-28%), 1% (95% CI, 1%-3%), 20% (95% CI, 17%-23%), 86% (95% CI, 79%-92%), and 100% (95% CI, 99%-100%), respectively. Supplemental Figures 1 to 5 illustrate their relevant forest plots. Heterogeneity was higher in the “Insufficient” and “Benign” categories compared with the other three categories.

### Diagnostic Accuracy of Breast FNA to Detect Malignancy With the Yokohama Reporting System

To assess the diagnostic accuracy of breast FNA for each scenario, we constructed sROC curves and calculated the pooled DOR for both aforementioned scenarios, using the extracted TN, TP, FN, and FP data (Supplemental Table 1).

**FIGURE 2:**
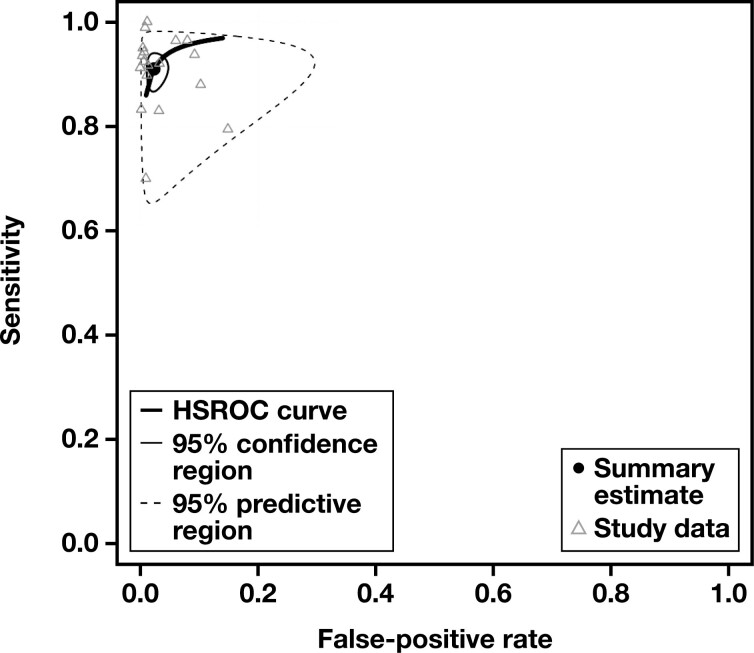
Summary ROC curve for detecting malignancy with breast fine-needle aspiration, using the Yokohama system. To construct this curve, “Suspicious” and “Malignant” interpretations from each study were considered cytologically positive, while carcinoma in situ and invasive cancer histology were the positive (malignant) reference standard. HSROC, hierarchical summary receiver operating characteristic.

**FIGURE 3:**
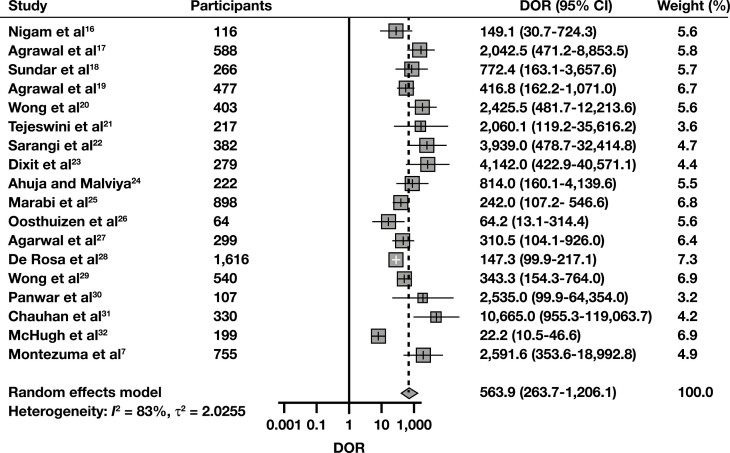
Diagnostic odds ratio (DOR) forest plot for detecting malignancy with breast fine-needle aspiration, using the Yokohama system. To construct this plot, “Suspicious” and “Malignant” interpretations from each study were considered cytologically positive, while carcinoma in situ and invasive cancer histology were the positive (malignant) reference standard.

**FIGURE 4:**
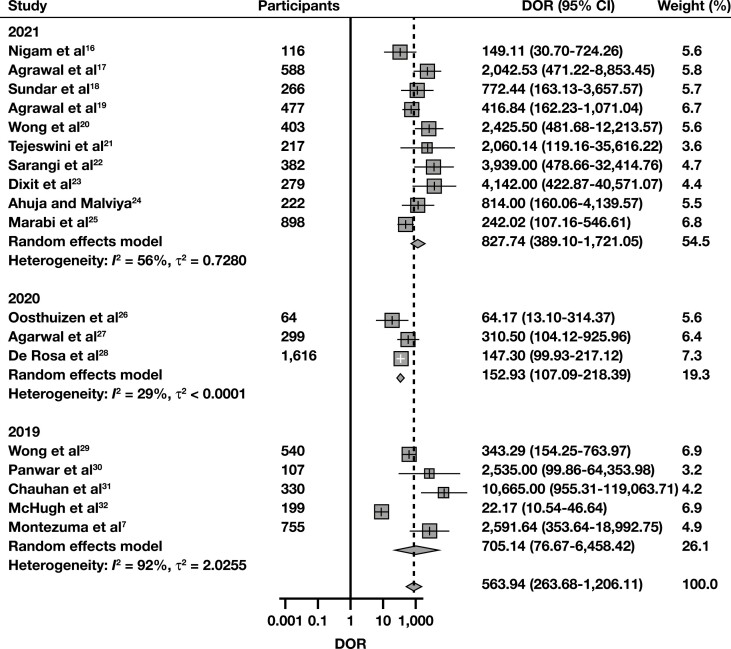
Diagnostic odds ratio (DOR) forest plot for detecting malignancy with breast fine needle-aspiration, with subgroup analysis regarding the year of publication. To construct this plot, “Suspicious” and “Malignant” Yokohama system interpretations from each study were considered cytologically positive, while carcinoma in situ and invasive cancer histology were the positive (malignant) reference standard.

**FIGURE 5:**
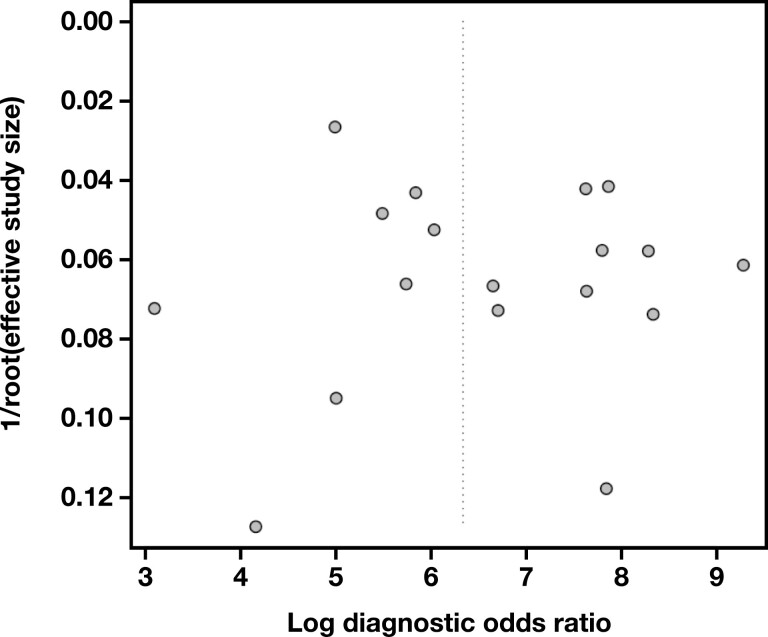
Funnel plot to evaluate the publication bias of our study.

The sROC curve for scenario 1, where “Suspicious” and “Malignant” interpretations were regarded as cytologically positive, is shown in **FIGURE 2**. The pooled AUC was 97.3%, indicating an excellent diagnostic accuracy. Sensitivity (SN) was 91% (95% CI, 87.6%-93.5%), and the false-positive rate (FPR) was 2.33% (95% CI, 1.30%-4.14%). The pooled DOR was 564 (95% CI, 264-1,206), also indicating a high level of diagnostic accuracy, while its forest plot is shown in **FIGURE 3**. To discover potential sources of heterogeneity, selected subgroup and sensitivity analyses were performed. When eligible studies were subgrouped regarding their year of publication, studies published in 2020 and 2021 exhibited a lower level of heterogeneity than the ones published in 2019 **FIGURE 4**. This might be due to the accumulation of experience using this new system through time. Subgroup analyses for the variables “study type” and “follow-up type” revealed that prospective studies, as well as studies that used both histology and clinical follow-up, showed lower levels of heterogeneity (Supplemental Figures 6 and 7). However, these analyses have a low power, as all of the above-mentioned subgroups were composed of just two studies each. A sensitivity analysis for the QUADAS-2 risk of bias category “study selection” was also performed and showed that the DOR was very similar between the two subgroups, yet this analysis also has a low power, as again only two studies comprised the high risk of bias subgroup (Supplemental Figure 8). Last, a funnel plot was constructed **FIGURE 5**, which did not reveal the presence of publication bias (*P* = .35).

**FIGURE 6:**
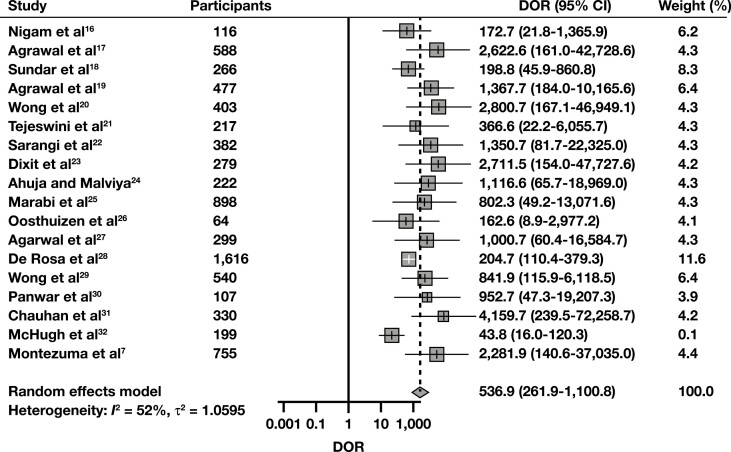
Diagnostic odds ratio (DOR) forest plot for detecting malignancy with breast fine-needle aspiration, using the Yokohama system. To construct this plot, only “Malignant” interpretations from each study were considered cytologically positive, while carcinoma in situ and invasive cancer histology were the positive (malignant) reference standard.

For scenario 2, when only “Malignant” interpretations were regarded as cytologically positive, the sROC was also constructed (Supplemental Figure 9). The pooled AUC was 87.4%, while the SN was 76.61% (95% CI, 70.1%-82.1%) and FPR was 0.75% (95% CI, 0.39%-1.42%). The pooled DOR was 537 (95% CI, 262-1,101), and its relevant forest plot is shown in **FIGURE 6**. Thus, even if the FPR was lower, overall diagnostic accuracy was lower when using scenario 2 rather than scenario 1.

## DISCUSSION

To our knowledge, this is the first systematic review and meta-analysis regarding the use of the IAC Yokohama system for reporting breast FNAs. Similar works have been published about other cytopathology reporting systems, such as the Bethesda System for Reporting Thyroid Cytopathology,^33^ the Milan System for Reporting Salivary Gland Cytopathology,^34^ the Paris System for Reporting Urinary Cytopathology,^35^ and the Papanicolaou System for Reporting Pancreatobiliary Cytopathology.^36^ First, we calculated the pooled ROM of each reporting category, using the extracted data from 18 studies published between 2019 and 2021. This could give a clearer picture to what we have known so far, as the ROM of each Yokohama system category has originally been reported^3^ as a range extracted from just two studies,^7,29^ rather than a pooled number from more reports published worldwide, such as in this meta-analysis. Compared with the baseline systematic review by Hoda and Brachtel,^37^ which combined data from 26 studies reporting their data before this new system’s official publication (January 1997 to December 2017), we found a lower ROM for the “Insufficient” (17% vs 30.3%), “Benign” (1% vs 4.7%), and “Atypical” (20% vs 51.5%) categories but a higher ROM for the “Suspicious” (86% vs 85.4%) and “Malignant” (100% vs 98.7%) reporting categories. We also assessed the diagnostic accuracy of breast FNA for the detection of malignancy when applying the Yokohama system by constructing sROCs and calculating the pooled DORs. When both “Suspicious” and “Malignant” interpretations were regarded as cytologically positive, the pooled SN was 91% (95% CI, 87.6%-93.5%) and FPR was 2.33% (95% CI, 1.30%-4.14%), while DOR was 564 (95% CI, 264-1,206). In contrast, when only “Malignant” interpretations were regarded as cytologically positive, the pooled FPR was lower (0.75%; 95% CI, 0.39%-1.42%) but at the expense of SN (76.61%; 95% CI, 70.05%-82.10%). Notably, one of the included studies reclassified their original diagnoses according to the IAC Yokohama system and compared the two systems. ROM dropped from 1.9% to 0.5% for the benign interpretations, whereas it increased from 83.0% to 89.2% and 96.7% to 100% for suspicious and malignant interpretations, respectively. Furthermore, SN, specificity, positive predictive value, negative predictive value, and accuracy increased, while the Yokohama system exhibited a significantly enhanced diagnostic performance (*P* < .001).^25^

The IAC Yokohama system emphasizes the significance of using ROSE whenever possible, as the latter decreases the inadequate and at the same time boosts the benign and malignant interpretations. Furthermore, it reduces patients’ anxiety and offers the opportunity to triage the material for ancillary studies or ask for a CNB.^2,3,38^ Three of the included studies in our review performed a separate analysis regarding the value of ROSE. Agrawal et al^17^ reported that the use of ROSE reduced their inadequate (6.8% to 1.8%; *P* < .001) and suspicious cytologic interpretations (4.4% to 1.6%; *P* = .010) at a significant level and also increased the overall diagnostic accuracy of the procedure, while Wong et al^20^ also found that ROSE significantly decreased their inadequate results (*P* < .0001). Notably, another study demonstrated that ROSE significantly reduced the percentage of cases interpreted as inadequate from 17.1% to 4% (*P* < .001) and atypical from 24.4% to 18.6%, although at no significant level (*P* = .1556), whereas it significantly increased the percentage of cases reported as malignant from 17.9% to 39% (*P* < .001).^29^ We believe the absence of ROSE in many of the cases included in this meta-analysis could have contributed to the higher ROM in the “Inadequate” and “Atypical” categories compared with what was originally reported by the Yokohama system team.^3^

CNB has largely replaced FNA in the evaluation of palpable and nonpalpable breast masses, as FNA has some disadvantages (eg, it cannot distinguish in situ from invasive carcinomas and also results in relatively higher inadequate diagnoses, especially in inexperienced hands), while the predictive testing for neoadjuvant therapies is traditionally performed on tissues.^3,6-8,39^ A meta-analysis pooled data from 12 studies and demonstrated that, whereas both CNB and FNA exhibited high diagnostic accuracies, CNB had a higher sensitivity and similar specificity with FNA when evaluating suspicious breast lesions.^40^ Moreover, another meta-analysis showed that CNB was also more sensitive than FNB for detecting metastasis in the axillary lymph nodes, but both were highly specific.^41^ Of interest, the authors of the IAC Yokohama system have emphasized the importance of a good FNA sampling and smearing technique, both largely dependent on the skill and experience of the radiologists and/or pathologists involved in the breast FNA process.^3^ Indeed, in centers with a large case volume following a multidisciplinary approach, breast FNA exhibits excellent diagnostic accuracy in both palpable and nonpalpable breast masses, which could even be higher than that of CNB, while the combination of FNA and CNB could be the most optimal strategy.^42,43^ Other studies have also shown promising results supporting the combination of FNA and CNB.^44,45^

Our systematic review has some important limitations. We observed high levels of heterogeneity, especially while pooling the ROM of “Insufficient” and “Benign” categories, also in the DOR analysis. In addition, most included studies (n = 16) were retrospective in nature and conducted in a single country. The extracted data did not allow us to assess the pooled ROM and diagnostic accuracy of breast FNA in selected subgroups (eg, females vs males, palpable vs nonpalpable lesions, presence vs absence of image guidance, and conventional vs liquid-based cytology). Last, regarding the publication bias analysis, this is expected to have a low power in our study, as the plot’s accuracy drops when the odds ratio moves away from 1.^46,47^

In conclusion, to our knowledge, this is the first meta-analysis regarding the use of the IAC Yokohama system for reporting breast FNAs. The implementation of this system seems to be successful so far. It assigns patients to a reporting category linked with a specific ROM and enhances the overall diagnostic accuracy of the FNA procedure to detect malignancy. However, results are still premature, and more data, especially from prospective studies, would be needed to fully unravel its value in modern clinical practice.

## Supplementary Material

aqac132_suppl_Supplementary_Material

## Notes

This article is available for CME credit. Go to academic.oup.com/ajcp/pages/journal_cme to see the latest articles. The complete catalog of journal CME courses can be found at store.ascp.org.
