# Supplementary material for: The Use of the International Academy of Cytology Yokohama System for Reporting Breast Fine-Needle Aspiration Biopsy: A Systematic Review and Meta-Analysis
Source: Am J Clin Pathol. 2022 Nov 12;159(2):138–45. doi: 10.1093/ajcp/aqac132 (PMC9891409; doi:10.1093/ajcp/aqac132)
Supplement: aqac132_suppl_Supplementary_Material [file aqac132_suppl_supplementary_material.docx]

**Table S1.** Diagnostic accuracy extracted data of the Yokohama system for reporting Breast FNA for both scenario 1 and scenario 2.

| **First author, year** | **Scenario 1** | | | | **Scenario 2** | | | |
| --- | --- | --- | --- | --- | --- | --- | --- | --- |
|  | **TN** | **FN** | **TP** | **FP** | **TN** | **FN** | **TP** | **FP** |
| Nigam, 2021 [[1]](https://paperpile.com/c/HuKPNQ/Ip4lz) | 61 | 9 | 44 | 2 | 62 | 14 | 39 | 1 |
| Agrawal N., 2021 [[2]](https://paperpile.com/c/HuKPNQ/JRhKh) | 336 | 19 | 231 | 2 | 338 | 51 | 199 | 0 |
| Sundar, 2021 [[3]](https://paperpile.com/c/HuKPNQ/mgWsg) | 176 | 9 | 79 | 2 | 176 | 27 | 61 | 2 |
| Agrawal S., 2021 [[4]](https://paperpile.com/c/HuKPNQ/NJm9H) | 109 | 13 | 348 | 7 | 115 | 28 | 333 | 1 |
| Wong YP., 2021 [[5]](https://paperpile.com/c/HuKPNQ/GyUWM) | 297 | 6 | 98 | 2 | 299 | 18 | 86 | 0 |
| Tejeswini, 2021 [[6]](https://paperpile.com/c/HuKPNQ/JMU9I) | 103 | 10 | 104 | 0 | 103 | 41 | 73 | 0 |
| Sarangi, 2021 [[7]](https://paperpile.com/c/HuKPNQ/1xGjg) | 273 | 7 | 101 | 1 | 274 | 31 | 77 | 0 |
| Dixit, 2021 [[8]](https://paperpile.com/c/HuKPNQ/5a2L7) | 218 | 3 | 57 | 1 | 219 | 8 | 52 | 0 |
| Ahuja, 2021 [[9]](https://paperpile.com/c/HuKPNQ/6UMKf) | 148 | 6 | 66 | 2 | 150 | 15 | 57 | 0 |
| Marabi, 2021 [[10]](https://paperpile.com/c/HuKPNQ/Njqyt) | 724 | 50 | 117 | 7 | 731 | 108 | 59 | 0 |
| Oosthuizen, 2020 [[11]](https://paperpile.com/c/HuKPNQ/i32mf) | 35 | 3 | 22 | 4 | 39 | 8 | 17 | 0 |
| Agarwal A., 2020 [[12]](https://paperpile.com/c/HuKPNQ/eU9ts) | 69 | 8 | 216 | 6 | 75 | 29 | 195 | 0 |
| De Rosa, 2020 [[13]](https://paperpile.com/c/HuKPNQ/FSdwo) | 453 | 70 | 1047 | 46 | 488 | 199 | 918 | 11 |
| Wong S., 2019 [[14]](https://paperpile.com/c/HuKPNQ/vxI0p) | 267 | 21 | 243 | 9 | 275 | 65 | 199 | 1 |
| Panwar, 2019 [[15]](https://paperpile.com/c/HuKPNQ/CKSs2) | 84 | 0 | 22 | 1 | 85 | 3 | 19 | 0 |
| Chauhan, 2019 [[16]](https://paperpile.com/c/HuKPNQ/pg7qD) | 237 | 1 | 90 | 2 | 239 | 9 | 82 | 0 |
| McHugh, 2019 [[17]](https://paperpile.com/c/HuKPNQ/RjFnO) | 103 | 16 | 62 | 18 | 116 | 27 | 51 | 5 |
| Montezuma, 2019 [[18]](https://paperpile.com/c/HuKPNQ/ZSYz6) | 521 | 39 | 194 | 1 | 522 | 73 | 160 | 0 |

Note: In scenario 1, “suspicious” and “malignant” Yokohama system interpretations from each study were considered as cytologically positive, whereas in scenario 2 only the “malignant” interpretations from each study were considered as cytologically positive. In both scenarios, carcinoma in situ and invasive cancer histology were considered as a positive (malignant) reference standard.

Abbreviations: TN, true negative; FN, false negative; TP, true positive; FP, false positive

**Table S2**. Risk of Bias of the studies included in the meta-analysis, according to the Quality Assessment of Diagnostic Accuracy Studies 2 (QUADAS-2) risk of bias tool.

| **First author, year** | **Risk of Bias** |  |  |  | **Applicability** |  |  |
| --- | --- | --- | --- | --- | --- | --- | --- |
|  | **Patient Selection** | **Index Test** | **Reference Test** | **Flow and Timing** | **Patient Selection** | **Index Test** | **Reference Test** |
| Nigam, 2021 [[1]](https://paperpile.com/c/HuKPNQ/Ip4lz) | L | L | U | L | L | L | L |
| Agrawal N., 2021 [[2]](https://paperpile.com/c/HuKPNQ/JRhKh) | L | L | U | L | L | L | L |
| Sundar, 2021 [[3]](https://paperpile.com/c/HuKPNQ/mgWsg) | L | L | U | L | L | L | L |
| Agrawal S., 2021 [[4]](https://paperpile.com/c/HuKPNQ/NJm9H) | L | L | U | L | L | L | L |
| Wong YP., 2021 [[5]](https://paperpile.com/c/HuKPNQ/GyUWM) | L | L | U | L | L | L | L |
| Tejeswini, 2021 [[6]](https://paperpile.com/c/HuKPNQ/JMU9I) | L | L | U | L | L | L | L |
| Sarangi, 2021 [[7]](https://paperpile.com/c/HuKPNQ/1xGjg) | H | L | U | L | H | L | L |
| Dixit, 2021 [[8]](https://paperpile.com/c/HuKPNQ/5a2L7) | L | L | U | L | L | L | L |
| Ahuja, 2021 [[9]](https://paperpile.com/c/HuKPNQ/6UMKf) | L | L | U | L | L | L | L |
| Marabi, 2021 [[10]](https://paperpile.com/c/HuKPNQ/Njqyt) | L | L | U | H | L | L | U |
| Oosthuizen, 2020 [[11]](https://paperpile.com/c/HuKPNQ/i32mf) | H | L | U | H | H | L | U |
| Agarwal A., 2020 [[12]](https://paperpile.com/c/HuKPNQ/eU9ts) | L | L | U | L | L | L | L |
| De Rosa, 2020 [[13]](https://paperpile.com/c/HuKPNQ/FSdwo) | L | L | U | L | L | L | L |
| Wong S., 2019 [[14]](https://paperpile.com/c/HuKPNQ/vxI0p) | L | L | U | L | L | L | L |
| Panwar, 2019 [[15]](https://paperpile.com/c/HuKPNQ/CKSs2) | L | L | U | L | L | L | L |
| Chauhan, 2019 [[16]](https://paperpile.com/c/HuKPNQ/pg7qD) | L | L | U | L | L | L | L |
| McHugh, 2019 [[17]](https://paperpile.com/c/HuKPNQ/RjFnO) | L | L | U | L | L | L | L |
| Montezuma, 2019 [[18]](https://paperpile.com/c/HuKPNQ/ZSYz6) | L | L | U | L | L | L | L |

Abbreviations: L, low; U, unclear; H, high

**Figure S1-S5**. Forest plots for the “Insufficient”, “Benign”, “Atypical”, Suspicious”, and “Malignant” categories of the Yokohama system for reporting Breast FNA


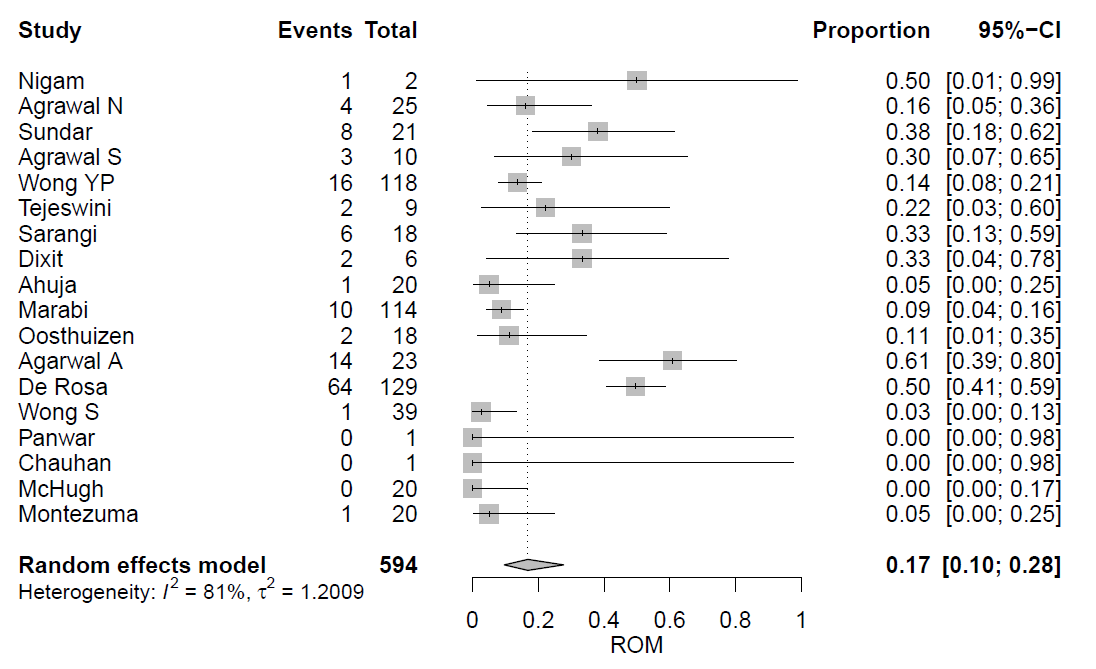


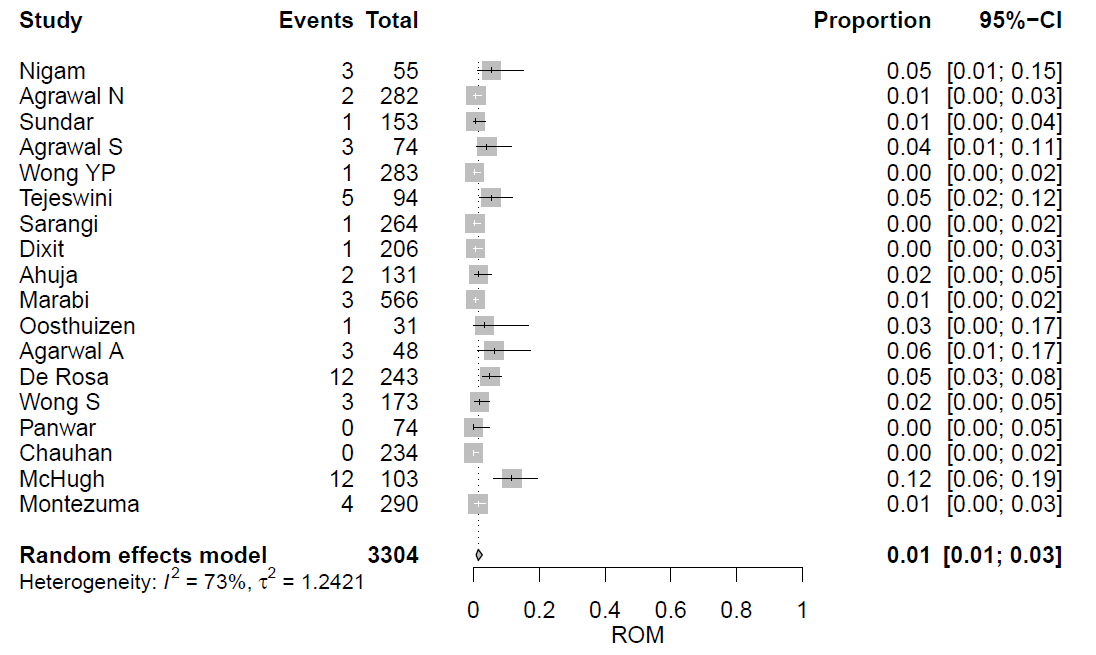


**
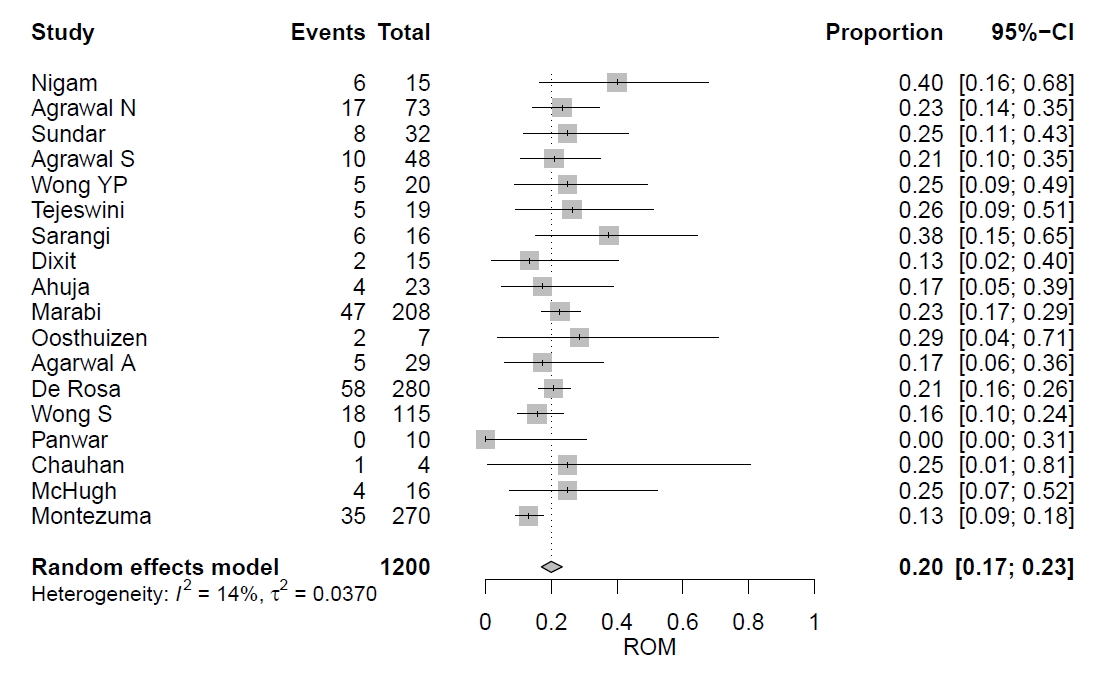
**

**
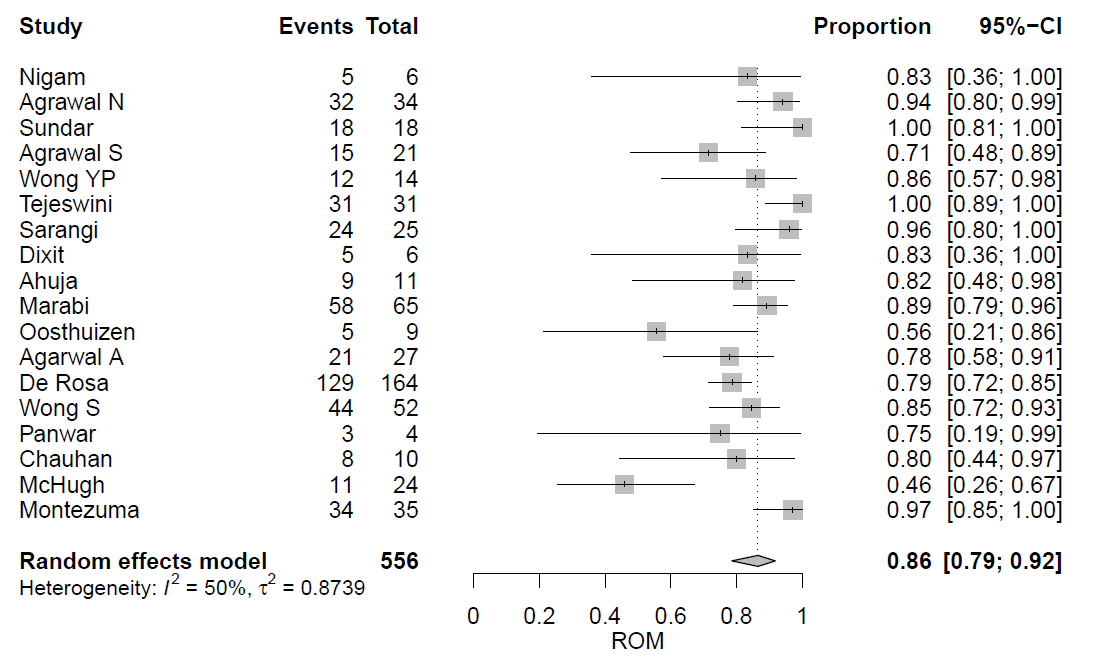
**

**
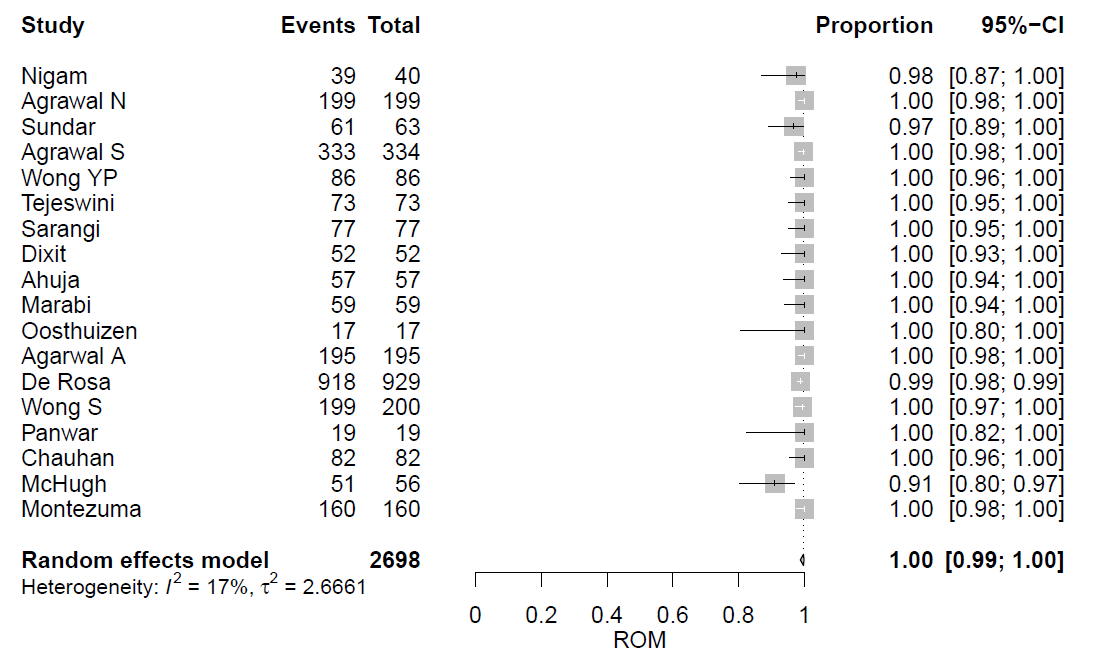
**

**Figures S6 and S7.** Diagnostic odds ratio (DOR) forest plot for detecting malignancy with breast fine needle-aspiration, with subgroup analyses regarding the variables “study type” and “follow-up type”. To construct these plots, “suspicious” and “malignant” Yokohama system interpretations from each study were considered as cytologically positive, while carcinoma in situ and invasive cancer histology as a positive (malignant) reference standard.

**
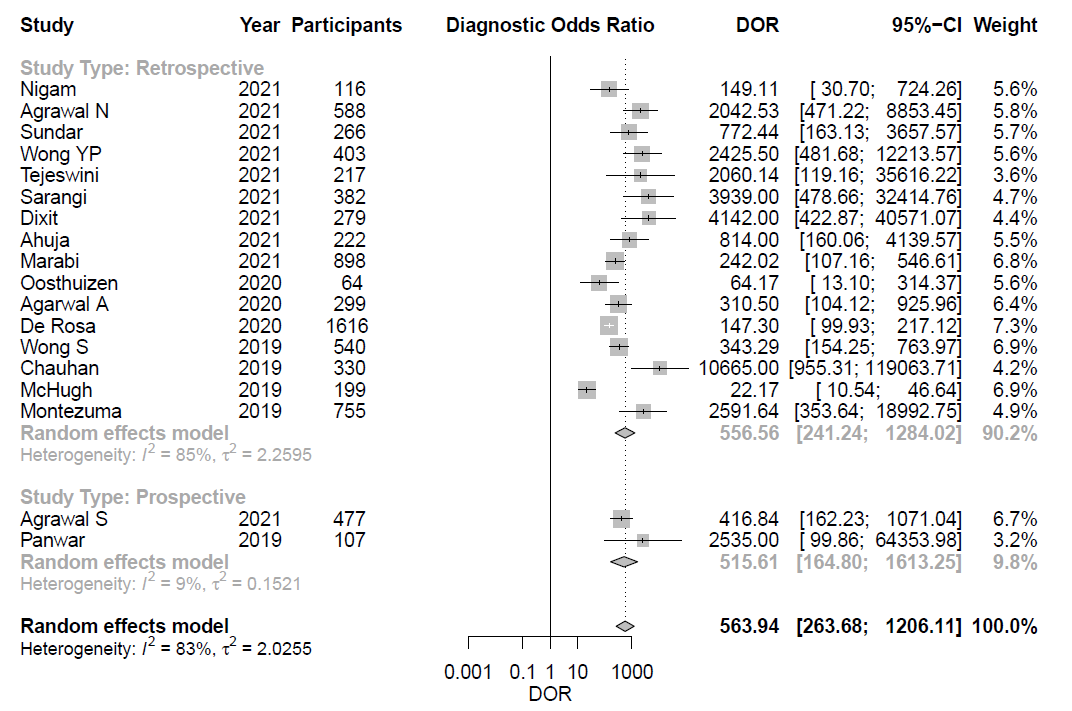
**

**
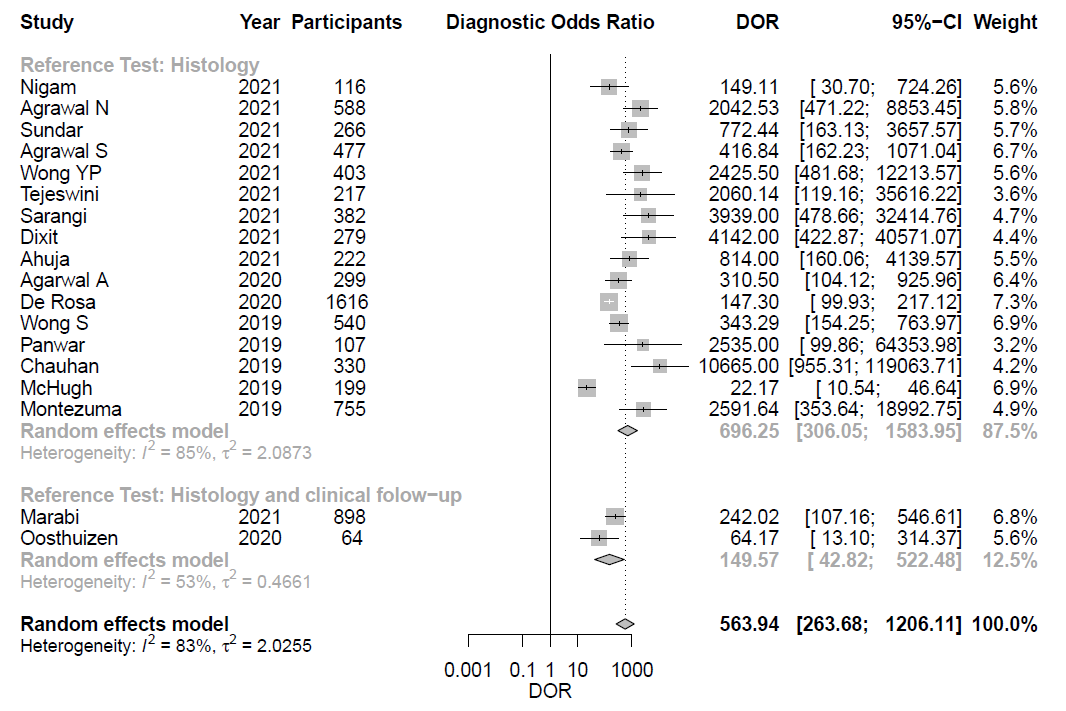
**

**Figure S8.** Diagnostic odds ratio (DOR) forest plot for detecting malignancy with breast fine needle-aspiration, with subgroup analysis regarding the category “study selection” of the QUADAS-2 risk of bias tool. To construct these plots, “suspicious” and “malignant” Yokohama system interpretations from each study were considered as cytologically positive, while carcinoma in situ and invasive cancer histology as a positive (malignant) reference standard.

**
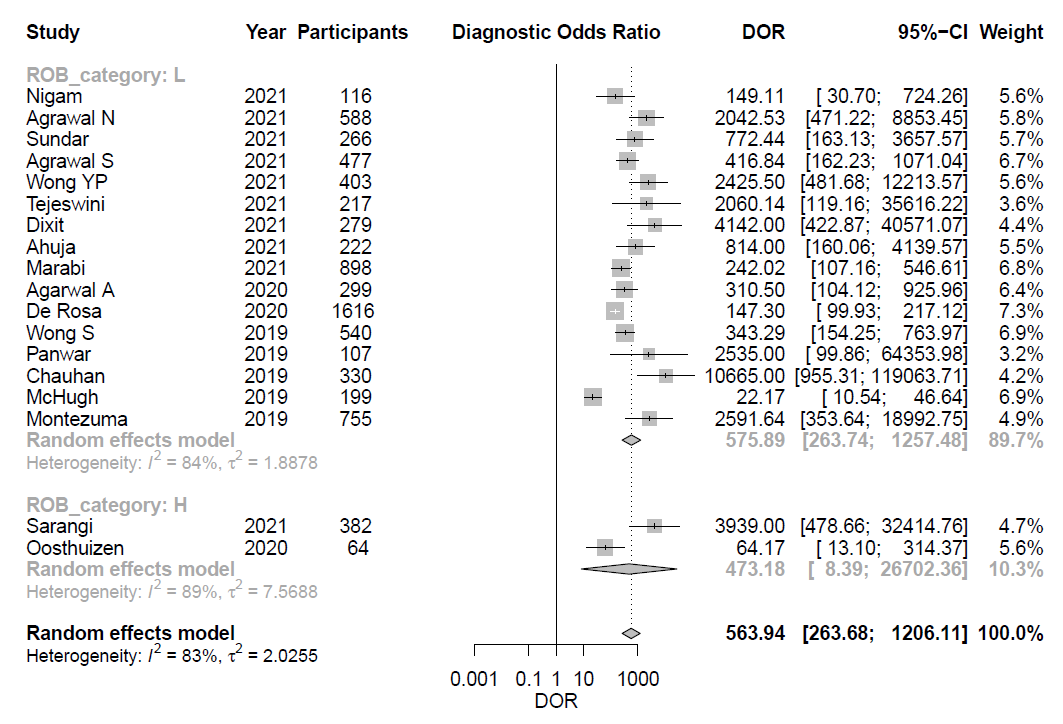
**

**Figure S9.** Summary ROC curve for detecting malignancy with breast fine-needle aspiration, using the Yokohama system. To construct this curve, only the “malignant” interpretations from each study were considered as cytologically positive, while carcinoma in situ and invasive cancer histology as a positive (malignant) reference standard.


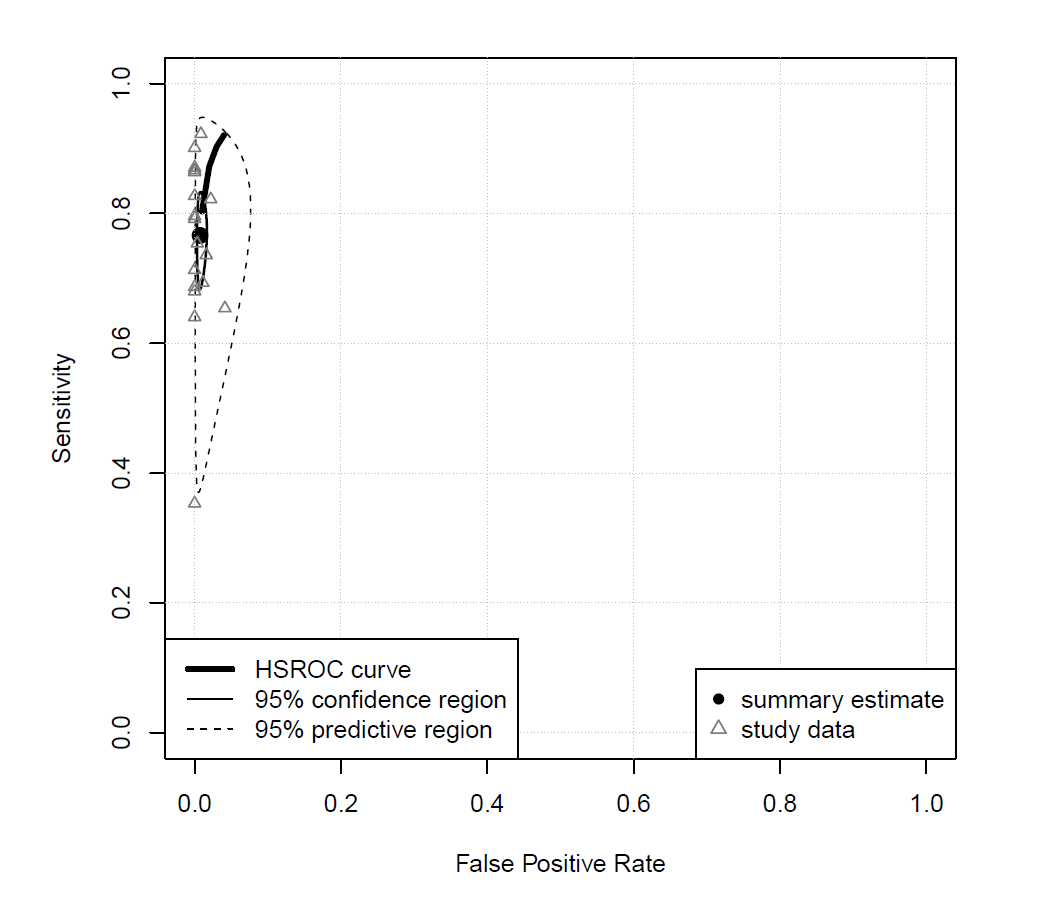


**Bibliography**

1 [Nigam JS, Kumar T, Bharti S, et al. The International Academy of Cytology standardized reporting of breast fine-needle aspiration biopsy cytology: A 2 year’s retrospective study with application of categories and their assessment for risk of malignancy. *Cytojournal* 2021; 18; 27.](http://paperpile.com/b/HuKPNQ/Ip4lz)

2 [Agrawal N, Kothari K, Tummidi S, et al. Fine-Needle Aspiration Biopsy Cytopathology of Breast Lesions Using the International Academy of Cytology Yokohama System and Rapid On-Site Evaluation: A Single-Institute Experience. *Acta Cytol.* 2021; 65; 463-477.](http://paperpile.com/b/HuKPNQ/JRhKh)

3 [Sundar PM, Shanmugasundaram S, Nagappan E. The role of the IAC Yokohama System for Reporting Breast Fine Needle Aspiration Biopsy and the ACR Breast Imaging-Reporting and Data System in the evaluation of breast lesions. *Cytopathology* 2022; 33; 185-195.](http://paperpile.com/b/HuKPNQ/mgWsg)

4 [Agrawal S, Anthony ML, Paul P, et al. Prospective evaluation of accuracy of fine-needle aspiration biopsy for breast lesions using the International Academy of Cytology Yokohama System for reporting breast cytopathology. *Diagn. Cytopathol.* 2021; 49; 805-810.](http://paperpile.com/b/HuKPNQ/NJm9H)

5 [Wong YP, Vincent James EP, Mohammad Azhar MAA, et al. Implementation of the International Academy of Cytology Yokohama standardized reporting for breast cytopathology: An 8-year retrospective study. *Diagn. Cytopathol.* 2021; 49; 718-726.](http://paperpile.com/b/HuKPNQ/GyUWM)

6 [Tejeswini V, Chaitra B, Renuka IV, et al. Effectuation of International Academy of Cytology Yokahama Reporting System of Breast Cytology to Assess Malignancy Risk and Accuracy. *J. Cytol.* 2021; 38; 69-73.](http://paperpile.com/b/HuKPNQ/JMU9I)

7 [Sarangi S, Rao M, Elhence PA, et al. Risk Stratification of Breast Fine-Needle Aspiration Biopsy Specimens Performed without Radiologic Guidance by Application of the International Academy of Cytology Yokohama System for Reporting Breast Fine-Needle Aspiration Cytopathology. *Acta Cytol.* 2021; 65; 483-493.](http://paperpile.com/b/HuKPNQ/1xGjg)

8 [Dixit N, Trivedi S, Bansal VK. A retrospective analysis of 512 cases of breast fine needle aspiration cytology utilizing the recently proposed IAC Yokohama system for reporting breast cytopathology. *Diagn. Cytopathol.* 2021; 49; 1022-1031.](http://paperpile.com/b/HuKPNQ/5a2L7)

9 [Ahuja S, Malviya A. Categorization of Breast Fine Needle Aspirates Using the International Academy of Cytology Yokohama System Along with Assessment of Risk of Malignancy and Diagnostic Accuracy in a Tertiary Care Centre. *J. Cytol.* 2021; 38; 158-163.](http://paperpile.com/b/HuKPNQ/6UMKf)

10 [Marabi M, Aphivatanasiri C, Jamidi SK, et al. The International Academy of Cytology Yokohama System for Reporting Breast Cytopathology showed improved diagnostic accuracy. *Cancer Cytopathol.* 2021; 129; 852-864.](http://paperpile.com/b/HuKPNQ/Njqyt)

11 [Oosthuizen M, Razack R, Edge J, et al. Classification of Male Breast Lesions According to the IAC Yokohama System for Reporting Breast Cytopathology. *Acta Cytol.* 2021; 65; 132-139.](http://paperpile.com/b/HuKPNQ/i32mf)

12 [Agarwal A, Singh D, Mehan A, et al. Accuracy of the International Academy of Cytology Yokohama system of breast cytology reporting for fine needle aspiration biopsy of the breast in a dedicated breast care setting. *Diagn. Cytopathol.* 2021; 49; 195-202.](http://paperpile.com/b/HuKPNQ/eU9ts)

13 [De Rosa F, Migliatico I, Vigliar E, et al. The continuing role of breast fine-needle aspiration biopsy after the introduction of the IAC Yokohama System For Reporting Breast Fine Needle Aspiration Biopsy Cytopathology. *Diagn. Cytopathol.* 2020; 48; 1244-1253.](http://paperpile.com/b/HuKPNQ/FSdwo)

14 [Wong S, Rickard M, Earls P, et al. The International Academy of Cytology Yokohama System for Reporting Breast Fine Needle Aspiration Biopsy Cytopathology: A Single Institutional Retrospective Study of the Application of the System Categories and the Impact of Rapid Onsite Evaluation. *Acta Cytol.* 2019; 63; 280-291.](http://paperpile.com/b/HuKPNQ/vxI0p)

15 [Panwar H, Ingle P, Santosh T, et al. FNAC of Breast Lesions with Special Reference to IAC Standardized Reporting and Comparative Study of Cytohistological Grading of Breast Carcinoma. *J. Cytol.* 2020; 37; 34-39.](http://paperpile.com/b/HuKPNQ/CKSs2)

16 [Chauhan V, Pujani M, Agarwal C, et al. IAC standardized reporting of breast fine-needle aspiration cytology, Yokohama 2016: A critical appraisal over a 2 year period. *Breast Dis.* 2019; 38; 109-115.](http://paperpile.com/b/HuKPNQ/pg7qD)

17 [McHugh KE, Bird P, Sturgis CD. Concordance of breast fine needle aspiration cytology interpretation with subsequent surgical pathology: An 18-year review from a single sub-Saharan African institution. *Cytopathology* 2019; 30; 519-525.](http://paperpile.com/b/HuKPNQ/RjFnO)

18 [Montezuma D, Malheiros D, Schmitt FC. Breast Fine Needle Aspiration Biopsy Cytology Using the Newly Proposed IAC Yokohama System for Reporting Breast Cytopathology: The Experience of a Single Institution. *Acta Cytol.* February 2019; 1-6.](http://paperpile.com/b/HuKPNQ/ZSYz6)
